# Predicting breast cancer prognosis based on a novel pathomics model through CHEK1 expression analysis using machine learning algorithms

**DOI:** 10.1371/journal.pone.0321717

**Published:** 2025-05-09

**Authors:** Chen Chen, Dan Gao, Huan Yue, Huijing Wang, Rui Qu, Xiaochi Hu, Libo Luo

**Affiliations:** 1 Breast and Thyroid Center, The First People’s Hospital of Zunyi (The Third Affiliated Hospital of Zunyi Medical University), Zunyi, Guizhou, China; 2 Clinical Laboratory, The First People’s Hospital of Zunyi (The Third Affiliated Hospital of Zunyi Medical University), Zunyi, Guizhou, China; CNR, ITALY

## Abstract

**Background:**

Checkpoint kinase 1 (CHEK1) is often overexpressed in solid tumors. Nonetheless, the prognostic significance of CHEK1 in breast cancer (BrC) remains unclear. This study used pathomics leverages machine learning to predict BrC prognosis based on CHEK1 gene expression..

**Methods:**

Initially, hematoxylin-eosin (H&E)-stained images obtained from The Cancer Genome Atlas Breast Invasive Carcinoma (TCGA-BRCA) were segmented using Otsu’s method. Further, the sub-image features were extracted using machine learning algorithms based on PyRadiomics, mRMRe, and Gradient Boosting Machine (GBM). The predicted CHEK1 expression levels were represented as the pathomics score (PS) and validated using the corresponding RNA-seq data. The prognostic significance of both CHEK1 and PS was evaluated using Kaplan-Meier (KM), and univariate and multivariate Cox regression. The model was assessed by comparing CHEK1 expression by immunohistochemistry (IHC) with PS in BrC tissue microarray (TMA).

**Results:**

A 633 × 10 sub-image set was eligible for training and a 158 × 10 set for validation. 1,488 features were extracted and 8 recursive feature elimination (RFE)-screened features were used to generate the model. A high PS was associated with CHEK1 overexpression, significantly correlating with survival outcomes, especially within 96 months post-diagnosis. Further, patients with high PS responded to anti-programmed cell death protein 1 (anti-PD-1) and anti-cytotoxic T lymphocyte antigen-4 (anti-CTLA4) treatments. In TMA validation, the IHC analysis estimated that high PS similarly predicted poorer prognosis and correlated with higher CHEK1 expression.

**Conclusions:**

The novel pathomics model reliably predicted CHEK1 expression using machine learning algorithms, which might provide potential clinical utility for prognosis and treatment guidance.

## Background

Breast cancer (BrC) has emerged as the most commonly diagnosed cancer, accounting for the leading cause of cancer-related deaths among women [1,2]. Despite the breakthroughs in terms of exploring origination and developing innovative treatment modalities, such as anti-human epidermal growth factor receptor 2 (anti-HER2) blockade, cyclin-dependent kinase 4/6 (CDK4/6) inhibitors, and antibody-drug conjugates, a certain proportion of patients often experience disease recurrence or progression, especially those at high risk [3–7]. Along this line, numerous prognostic biomarkers have been developed for BrC, including estrogen receptor (ER), progesterone receptor (PR), HER2, Ki67, carcinoembryonic antigen (CEA), and cancer antigen 15–3 (CA15–3) [8–10]. Nevertheless, the performance of these prognostic biomarkers in real-world populations has been shown to be sub-optimal, emphasizing the requirement for further exploration of innovative prognostic biomarkers and enabling personalized risk stratification and individualized precision therapy.

Checkpoint kinase 1 (CHEK1) is a serine/threonine-specific protein kinase encoded by the CHEK1 gene located on Chromosome 11q24.2 [11,12]. CHEK1, a central component of genome surveillance pathways, is involved in various biological processes, including cell cycle regulation, cell survival, deoxyribonucleic acid (DNA) repair, transcription, egg production, embryo development, human immunodeficiency virus (HIV) response, and somatic cell viability [13–16]. CHEK1 overexpression has often been observed in various solid tumours, including BrC [17]. Moreover, the prognostic significance of CHEK1 has been explored in various cancers, such as melanoma [18], lung adenocarcinoma [19], hepatocellular carcinoma [20,21], and BrC [17]. The potential anti-cancer effects of CHEK1 inhibitors are currently being evaluated in clinical trials as a single therapeutic agent or as potential enhancers of chemotherapeutic agents in combination [22,23]. Despite the success, the predictable measurement of CHEK1 in the clinical practice for determining the prognosis information is currently hindered by various challenges of sample collection, expensiveness, time-consuming, operator proficiency, and reagent heterogeneity. Recent studies have emphasized the prognostic value of CHEK1 in breast cancer [24–26]. However, these studies primarily focus on high-throughput data analysis and immunohistochemical studies, which present challenges in clinical application due to the reasons previously mentioned. On one end, pathomics is an emerging field that integrates pathology images and omics data to develop certain predictive models for improving disease diagnosis and prognosis [27–29]. On the other end, Machine learning is a subfield of AI that involves developing algorithms capable of learning from and making predictions based on data. While some machine learning methods can operate with relatively smaller amounts of data, most effective models require substantial training data and careful programming to optimize performance and accuracy [30–32]. Along this line, a pathomics model based on machine learning can predict the gene expression levels from the hematoxylin-eosin (H&E)-stained images, which are essential for pathological diagnosis [33–35]. These innovative models analyze shape-based (such as volume and surface area), intensity-based (such as mean, variance, and skewness), and texture-based (such as entropy, energy, and homogeneity) features to make predictions [36]. In this study, we introduce a novel approach that leverages a pathomics model combined with machine learning algorithms to predict CHEK1 expression from H&E-stained images. This approach not only addresses the limitations of conventional prognostic biomarkers but also provides a new dimension of integrating digital pathology with molecular data, potentially enhancing the precision of clinical prognosis and treatment guidance.

Motivated by these considerations, this study proposed an innovative approach to predicting CHEK1 expression from H&E-stained images using a pathomics model trained with machine learning algorithms. Initially, the H&E-stained images obtained from the Cancer Genome Atlas Breast Invasive Carcinoma (TCGA-BRCA) dataset were segmented using Otsu’s method [37]. Subsequently, the sub-image features were extracted and evaluated using machine learning algorithms based on PyRadiomics, mRMRe, and Gradient Boosting Machine (GBM). These bioinformatics tools were used to explore the underlying molecular implications of the predicted pathomics scores (PS). Finally, the clinical applicability of the pathomics model was evaluated using the BrC tissue microarray (TMA) slides.

## Methods

### Patients

**Fig 1:**
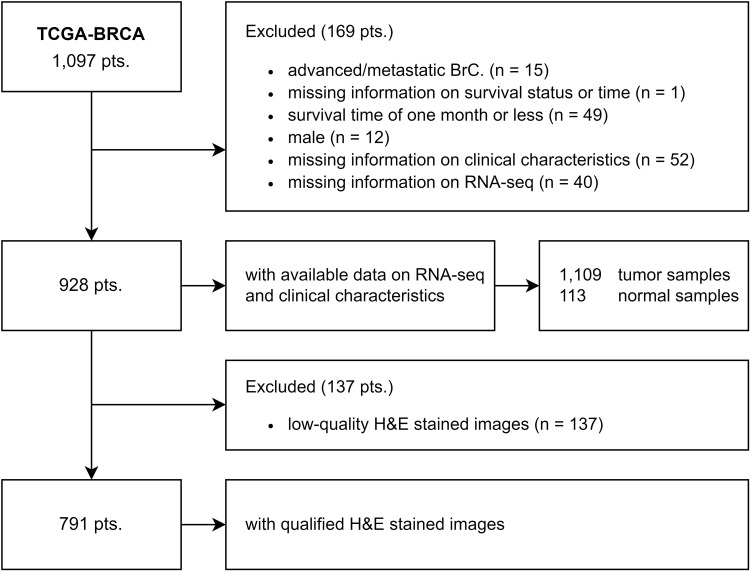
The flowchart included patients, samples and images. A patient may have multiple tumor samples, and not all patients necessarily have normal samples available. Images showing obvious contamination, blurring, or blank areas exceeding 50% are defined as low-quality. Abbreviations: pts., patients; BrC., breast cancer.

The ribonucleic acid (RNA)-seq data, clinical features, and H&E-stained images were obtained from TCGA-BRCA datasets and retrospectively analyzed (Fig 1) [38–41]. Among the 1,224 total patients downloaded from TCGA, 1,111 were patients, and 113 were normal or paracancerous samples. Following clinical data curation, 1,097 patients with available clinical information were identified. Of these, 928 tumor patients were eligible for gene expression analysis and survival analysis. In addition, 791 patients were found eligible for the pathomics analysis with qualified H&E-stained images.

The following clinical characteristics were included for analysis: age (<60 years and ≥ 60 years), T stage (T1, T2, and T3/T4), N stage (N0 and N1/N2/N3/Nx), M stage (M0 and M1/Mx), ER status (negative and positive), PR status (negative and positive), HER2 status (negative, positive, and unknown), histological type (infiltrating ductal carcinoma, IDC, infiltrating lobular carcinoma, ILC, and others), margin status (negative, positive, close, and unknown), radiotherapy status (no and yes), chemotherapy status (negative and positive), survival status (living and dead), and survival time (months). Breast cancer subtypes were classified as follows: Luminal A (ER-positive, PR-positive, HER2-negative), Luminal B (ER-positive, PR-negative or HER2-positive), Basal (ER-negative, PR-negative, HER2-negative), HER2 (HER2-positive, ER-negative, PR-negative), and others (including rare and mixed subtypes). Considering these attributes, 306 patients who met the notified criteria were deemed ineligible and were excluded: 1) advanced or metastatic BrC upon initial diagnosis; 2) unavailable data on survival status or time; 3) documented survival time of one month or less; 4) male patients; 5) missing information on the above-mentioned clinical characteristics; 6) unavailable RNA-seq data; and 7) low-quality or missing H&E-stained images.

### Gene expression

TCGA data (https://genome-cancer.ucsc.edu/) and Genotype-Tissue Expression (GTEx) data were downloaded from the UCSC Xena browser (https://xenabrowser.net). Differential expression analysis of CHEK1 was performed using the R package limma, with statistical significance determined by a p-value ≤ 0.05. The RNA-seq data extracted from the TCGA-BRCA dataset normalized to log2(FPKM+1) were used to evaluate the gene expression levels in both tumor and normal tissues [42]. The R package “survminer” (**https://cran.r-project.org/web/packages/survminer/index.html**) was utilized to determine the cut-off value for differentiating between high- and low-CHEK1 expressions. The recruited patients’ data were categorized into “CHEK1-high” and “CHEK1-low” groups based on the cut-off value (2.2443). Further, the Toil software was applied to compare the differences in the expression levels of CHEK1 between tumor and normal tissues, following the recommended workflow as stated in the literature [43].

### Survival analysis

The Kaplan-Meier (KM) survival curves were plotted using the R package “survival” (https://cran.r-project.org/web/packages/survival/index.html), illustrating survival differences among different groups of patients. The R package “survminer” was utilized to visualize survival data. It should be noted that the landmark analysis was performed using the R packages “jsKM” (https://cran.r-project.org/web/packages/jskm/index.html) and “survival” to minimize the impact of baseline risk changes on survival. The landmark KM curves were plotted for different time segments (24, 36, 48, 60, 72, 84, 96, 108, 120, 132, and 144 months after diagnosis) [44–46]. The time from the first diagnosis up to each time point was defined as “early-stage”. Contrarily, the time from each time point to the end of the follow-up was characterized as “late-stage”. Finally, the log-rank test was used to assess the difference in the survival rates among the treatment groups.

The Cox proportional hazard regression model was applied to explore the prognostic significance of clinical characteristics on survival outcomes. The single-factor (univariate) and multiple-factor (multivariate) Cox regression analyses were performed using the R packages “survival” (https://cran.r-project.org/web/packages/survival/index.html) and “forestplot” (**https://cran.r-project.org/web/packages/forestplot/index.html**). The univariate Cox regression analysis was performed to demonstrate the effect of CHEK1 expression or PS (high *vs.* low) on patient prognosis in different subgroups, using the R packages “survival,” “forestplot,” and “cmprsk” (https://cran.r-project.org/web/packages/cmprsk/index.html). Further, the interaction between CHEK1 or pathological score and other variables was analyzed using the likelihood ratio test.

### Pathomics model

#### Image acquisition and segmentation.

The H&E-stained images of formalin- and paraffin-embedded tumor tissues were downloaded from the TCGA database (**https://tcga-data.nci.nih.gov/tcga/%EF%BC%89**) in the svs format, with a maximum magnification of 20 × or 40× [47,48]. The extracted H&E-stained images that were evaluated manually, without obvious artefacts, were selected for subsequent analysis. The OTSU algorithm (**https://opencv.org**) was used to divide images into two parts: the unwanted background and the required tissue area (S1 Fig) [49,50]. Subsequently, the 40 × images were divided into multiple 1024x1024-pixel sub-images. Similarly, the 20 × images were divided into multiple 512x512-pixel sub-images, which were then magnified to 1024x1024 pixels. Furthermore, these sub-images were reviewed by pathologists to exclude images with poor quality, such as contamination, blurring, or blank areas exceeding 50%. Eventually, the sub-images (n = 10) of each patient were randomly selected for further analysis [47,48].

#### Image feature extraction and screening.

Image feature extraction and screening were performed using the open-source Python package PyRadiomics (https://pyradiomics.readthedocs.io/en/latest) with standardized sub-images [36]. This process involved calculating various radiomic features, including shape, first-order statistics, and texture descriptors. For instance, shape features can be defined mathematically using parameters such as volume *V*, surface area *SA*, and compactness *C*, which are calculated based on the three-dimensional representation of the image. Specifically, the volume *V* is calculated as the product of the longest axis and the product of the other two axes, while the surface area *SA* is calculated as the sum of the products of the axes with their corresponding semi-axes. The ompactness *C*, on the other hand, is calculated as the volume divided by the surface area.

Pathological features of each sample were determined by calculating the average values from ten random sub-images, ensuring robust feature representation. The dataset was then divided into training and validation sets using the R package “CBCgrps” (https://cran.r-project.org/package=CBCgrps). Z-score standardization was performed on the training set’s pathological features, defined as:


$$z=\frac{\left(X-\mu \right)}{\sigma }$$

where *μ* is the mean and *σ* is the standard deviation of the training set. The validation set was standardized using the training set’s mean and standard deviation via the R package “caret” (**https://cran.r-project.org/web/packages/caret/index.html**). This approach ensures that the model’s performance is evaluated on data that is representative of the overall dataset.

Statistical differences in clinical characteristics between the training and validation sets were assessed using t-tests. The optimal feature subset was selected using the R packages “mRMRe” (https://cran.r-project.org/web/packages/mRMRe/index.html) and “caret”(https://cran.r-project.org/web/packages/caret/index.html) utilizing the Maximum Relevance, Minimum Redundancy (mRMR) algorithm defined as:


$$mRMR=max\sum_{𝕚=1}^{n}I(X_{i};Y)-\lambda \sum i\neq jI(X_{i};X_{j})$$

and Recursive Feature Elimination (RFE), which systematically removes the least important features based on their contribution to the model.

In detail, the mRMR algorithm iteratively selects the feature with the maximum relevance to the target variable while minimizing its redundancy with other features. The calculation of the mutual information $I(X_{i};Y)$ is based on the Kullback-Leibler distance, which measures the amount of information shared between two random variables. The RFE algorithm, on the other hand, is a wrapper method that operates by recursively removing the feature with the least importance in the current model, thereby refining the feature subset.

Additionally, the impact of the selected features on the model’s performance was assessed by performing a feature importance analysis using the R package “randomForest” (https://cran.r-project.org/web/packages/randomForest/).

#### Model generation, evaluation, and validation.

In this study, we employed the Gradient Boosting Machine (GBM) algorithm to generate a pathomics model for predicting gene expression from screened pathological features in the training set. GBM is a robust ensemble learning technique that constructs a predictive model through the sequential combination of weak classifiers. Each classifier is trained to correct the errors made by the previous one. The mathematical formulation of the GBM model can be expressed as follows:


$$F\left(x\right)=F_{0}\left(x\right)+\sum_{m=1}^{M}\gamma_{m}h_{m}\left(x\right)$$

where $F_{0}\left(x\right)$ is the initial model, $h_{m}\left(x\right)$ represents the weak classifier, and $\gamma_{m}$ denotes the contribution of the m-th classifier. The objective is to minimize the loss function L(Y,F(x)) over the training data, where 𝒴 is the true label.

For model training, we optimized several hyperparameters, including: Learning Rate (η): This parameter controls the contribution of each weak learner, the optimal value was set to 0.05. Number of Trees (n_estimators): The total number of weak classifiers to be combined, the total number of weak classifiers to be combined. We determined that the optimal value was 300. Maximum Depth (max_depth): This parameter determines the maximum depth of each tree, the optimal value was selected as 6 to prevent overfitting. Minimum Samples Split (min_samples_split): The minimum number of samples required to split an internal node, the optimal value was set to 5. To ensure the robustness of our model, we conducted extensive model evaluation and validation. We used 10-fold cross-validation on the training set to tune the hyperparameters and assess the model’s performance. This method involves dividing the training data into 10 subsets, iteratively using 9 for training and 1 for validation, and cycling through all subsets. The model’s predictive performance was evaluated using key metrics such as accuracy, precision, recall, and the F1-core. Additionally, an independent validation set was used to further validate the model’s generalizability. Additionally, an independent validation set was used to further validate the model’s generalizability. The performance of the pathomics model was rigorously evaluated using several R packages, including “pROC” (https://cran.r-project.org/web/packages/pROC/index.html) for ROC curve analysis, “ResourceSelection” (https://cran.r-project.org/web/packages/ResourceSelection/index.html) for calibration assessment, “rms” (https://cran.r-project.org/web/packages/rms/index.html) for regression modeling, and “rmda” (https://cran.r-project.org/web/packages/rmda/index.html)for decision analysis. Specifically, we utilized the calibration curve and the Hosmer-Lemeshow goodness-of-fit test to assess model calibration. Additionally, the Brier score was computed to evaluate predictive accuracy, with lower scores indicating more reliable predictions.

To illustrate the clinical applicability of the model, we conducted Decision Curve Analysis (DCA) [51], which assesses the net benefits of the predictive model across a range of threshold probabilities. The model was generated using the training image set derived from H&E-stained images based on TCGA-BRCA and subsequently validated with a corresponding validation image set as well as scanned images from H&E-stained TMA slides.

### Gene set enrichment analysis (GSEA)

GSEA was performed using the R package “clusterProfiler” (https://www.bioconductor.org/packages/release/bioc/html/clusterProfiler.html) following the official GSEA guidelines (**https://software.broadinstitute.org/cancer/software/gsea/wiki/index.php/Main_Page**) [52,53]. The pre-defined gene sets were obtained from the Molecular Signatures Database (MSigDB; http://software.broadinstitute.org/gsea/msigdb). Further, the differences in gene expression profiles between the PS-high and PS-low groups were compared using GSEA with the Hallmark gene sets (h.all.v7.5.1.symbols.gmt) and the Kyoto Encyclopedia of Genes and Genomes (KEGG) sets (c2.cp.kegg.v7.5.1.symbols.gmt). Accordingly, the top 5 positively enriched signalling pathways were selected for further analysis. It should be noted that a P-value of less than 0.05 and a false discovery rate (FDR) of less than 0.25 were considered statistically significant.

### Immune composition analysis

The ImmuCellAI database (**http://bioinfo.life.hust.edu.cn/ImmuCellAI/#!**) was employed to determine variations in the immune cell infiltration by uploading the gene expression matrix of selected patients [54,55]. The Wilcoxon rank sum test was conducted using the R package “limma” (**https://bioconductor.org/packages/release/bioc/html/limma.html**) to analyze the differences in the immune gene expression and immune cell infiltration among different groups. The Tumor Immune Dysfunction and Exclusion (TIDE) computational framework (**http://tide.dfci.harvard.edu**) was applied to predict the potential response to anti-programmed cell death protein 1 (anti-PD-1) and anti-cytotoxic T lymphocyte antigen-4 (anti-CTLA4) treatments. Notably, higher TIDE prediction scores were not only associated with inferior treatment efficacy but also with worse survival outcomes. Finally, the TIDE prediction results were statistically analyzed and visualized using the R package “limma”.

### Tumor mutational burden analysis

Somatic mutation information for BRCA was obtained from the TCGA (https://genome-cancer.ucsc.edu/). Using Perl scripts, somatic mutation data were extracted, and the TMB score for each sample was calculated by dividing the number of mutations by the exome length. Concurrently, the expression of the target gene CHEK1 was extracted. The correlation between TMB scores and CHEK1 expression was analyzed using the R package “limma” (https://bioconductor.org/packages/release/bioc/html/limma.html).

### TMA

The human BrC TMA slides (ZL-BRCsur1801) were procured from WEIAO Biotechnology Co. Ltd. (Shanghai, China). Each slide contained a total of 90 pairs of BrC and corresponding normal tissue samples from women. Accordingly, the available information on clinical characteristics for analysis included age, tumor, node, metastasis (TNM) stage, ER status, PR status, HER2 status, KI67 levels, histologic type, histologic grade, and overall survival (OS) time. The H&E-stained images on TMA slides were processed and analyzed using the pathomics model to obtain a PS value.

### Immunohistochemical (IHC) analysis

The IHC analysis was performed using the CHEK1 polyclonal antibody (25887–1-AP, Proteintech, Rosemont, USA) at a concentration of 1:50. Further, the histochemistry score (HS) was determined by evaluating the percentage and intensity of positive CHEK1 staining on the TMA slides using the AI-driven Visiopharm^®^ image analysis software [56,57]. Specifically, the HS is calculated by combining the percentages of strongly stained nuclei (SSN), moderately stained nuclei (MSN), and weakly stained nuclei (WSN) using the following equation: H-Score = 1 × WSN + 2 × MSN + 3 × SSN [58]. Eventually, the results ranged from 0 to 300, with higher values indicating a stronger overall positive intensity. The cut-off value (141.41) was determined using the R package “survminer” to distinguish between high HS and low HS levels.

## Results

### CHEK1 overexpression is associated with poor prognostic outcomes

To elucidate the clinical significance of CHEK1 as a potent biomarker across various cancer types, we analyzed CHEK1 expression using RNA-seq data from 33 cancer types available through the UCSC Xena browser. Our analysis revealed that CHEK1 expression was consistently higher in tumor tissues compared to normal tissues across a range of cancer types, including BLCA, BRCA, COAD, and LIHC, with statistically significant differences (P < 0.001). However, no significant differences were observed in PCPG, SARC, and THYM cancers (S2 Fig). These findings suggest that CHEK1 may play a critical role in tumor prognosis and progression, prompting further investigation in breast cancer.

**Fig 2:**
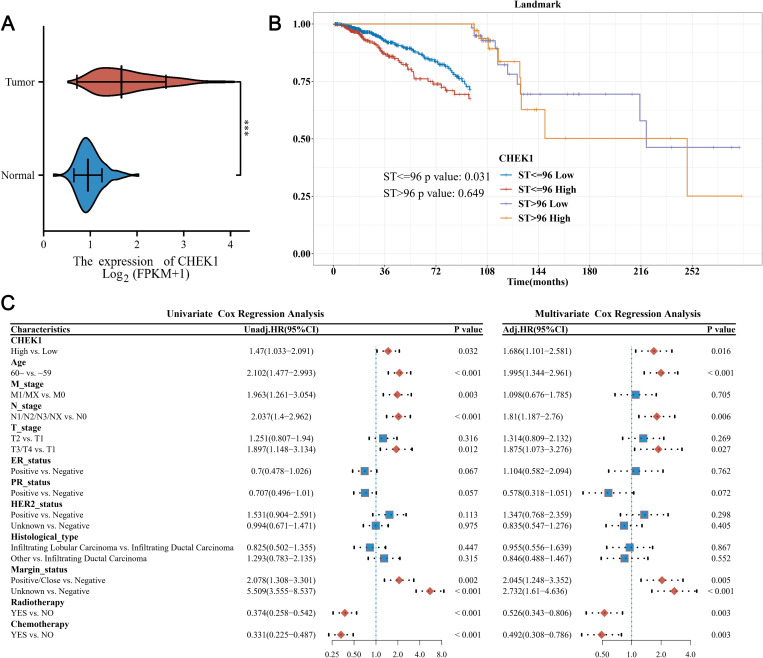
Evaluation of the prognostic significance of CHEK1 in BrC using the TCGA-BRCA dataset. A) The CHEK1 expression of BrC and normal breast tissues based on TCGA-BRCA RNA-seq data. B) The KM curves of OS rate based on CHEK1 expression in BrC tissues using 96 months post-diagnosis as the landmark. C) The univariate and multivariate Cox regression analyses of potential risk factors for OS. An HR value greater than 1 is a risk factor, while an HR value less than 1 is a protective factor. Abbreviations: BrC., breast cancer; *, P < 0.001; m, months; OS, overall survival; HR, hazard ratio.

The RNA-seq data presenting CHEK1 expression for a total of 928 patients were extracted from the TCGA-BRCA dataset. Based on the cut-off value of 2.2443, the recruited patients’ data were classified into “CHEK1-high” with a total of 619 patients and “CHEK1-low” with a total of 309 patients. Table 1 illustrates the characteristics of these patients’ data. The results indicated statistical differences (P < 0.05) in the CHEK1-high vs. CHEK1-low groups concerning the attributes of age, T stage, ER status, histological type, and chemotherapy status. According to the RNA-seq expression, the median expression of CHEK1 was 1.665 data in the log2(FPKM+1) format (with the 25^th^ percentile of 1.260 and the 75^th^ percentile of 2.212) in the tumor tissue samples. In contrast, the median expression of CHEK1 was 0.946 (0.817–1.118) in the normal tissue samples. It should be noted that the difference between the two (tumor and normal tissue samples) groups was significant (P < 0.001, Fig 2A). Analysis of CHEK1 expression levels across different breast cancer subtypes, including triple-negative breast cancer (Basal), hormone receptor-positive subtypes (Luminal A and Luminal B), and HER2-positive subtypes, revealed significant overexpression of CHEK1 in all subtypes compared to healthy controls (S3 Fig).

**Table 1:** Baseline Characteristics of Patients in the CHEK1-low and CHEK1-high Groups.

Variables	Total (n = 928)	CHEK1-Low (n = 619)	CHEK1-High (n = 309)	p
**Age, n (%)**				
<60	506 (55)	310 (50)	196 (63)	< 0.001
≥60	422 (45)	309 (50)	113 (37)	
**T Stage e, n (%)**				
T1	250 (27)	184 (30)	66 (21)	< 0.001
T2	528 (57)	325 (53)	203 (66)	
T3/T4	150 (16)	110 (18)	40 (13)	
**N Stage, n (%)**				
N0	430 (46)	276 (45)	154 (50)	0.149
N1/N2/N3/NX	498 (54)	343 (55)	155 (50)	
**M Stage, n (%)**				
M0	771 (83)	503 (81)	268 (87)	0.045
M1/MX	157 (17)	116 (19)	41 (13)	
**ER Status, n (%)**				
Negative	210 (23)	61 (10)	149 (48)	< 0.001
Positive	718 (77)	558 (90)	160 (52)	
**PR Status, n (%)**				
Negative	298 (32)	120 (19)	178 (58)	< 0.001
Positive	630 (68)	499 (81)	131 (42)	
**HER2 Status, n (%)**				
Negative	493 (53)	330 (53)	163 (53)	0.796
Positive	140 (15)	90 (15)	50 (16)	
Unknown	295 (32)	199 (32)	96 (31)	
**Histological Type, n (%)**				
Infiltrating Ductal Carcinoma	655 (71)	390 (63)	265 (86)	< 0.001
Infiltrating Lobular Carcinoma	185 (20)	166 (27)	19 (6)	
Other	88 (9)	63 (10)	25 (8)	
**Margin Status, n (%)**				
Negative	778 (84)	513 (83)	265 (86)	0.286
Positive/Close	99 (11)	73 (12)	26 (8)	
Unknown	51 (5)	33 (5)	18 (6)	
**Chemotherapy, n (%)**				
No	406 (44)	299 (48)	107 (35)	< 0.001
Yes	522 (56)	320 (52)	202 (65)	
**Radiotherapy, n (%)**				
No	442 (48)	298 (48)	144 (47)	0.709
Yes	486 (52)	321 (52)	165 (53)	

After diagnosis, the timepoint of 96 months was identified as the optimal landmark for evaluating the significance of CHEK1 expression in BrC cases (Fig 2B and S4 Fig). Briefly, overexpression of the CHEK1 gene could be significantly associated (P = 0.031) with the poor OS in the early-stage of BrC cases within 96 months after diagnosis. Nonetheless, the prognostic outcomes were similar in the late-stage, *i.e.,* later than 96 months after diagnosis, regardless of the CHEK1 expression levels in the respective groups. CHEK1 overexpression was found to be a risk factor for OS rates in both the univariate (HR = 1.47, 95% CI: 1.033–2.091, P = 0.032) and multivariate (HR = 1.686, 95% CI: 1.101–2.581, P = 0.016) analyses (Fig 2C). The subgroup analysis and interaction tests on baseline characteristics were performed, showing significant differences between the two groups (S6 Fig). The vile effect of CHEK1 overexpression on OS rate was independent of age (interaction test P = 0.687), T stage (P = 0.788 for T2 *vs.* T1 and P = 0.577 for T3/T4 *vs.* T1), ER status (P = 0.735), histological type (P = 0.127 for ILC *vs.* IDC and P = 0.805 for others *vs.* IDC), and chemotherapy status (P = 0.077).

### CHEK1 predicting pathomics model

**Fig 3:**
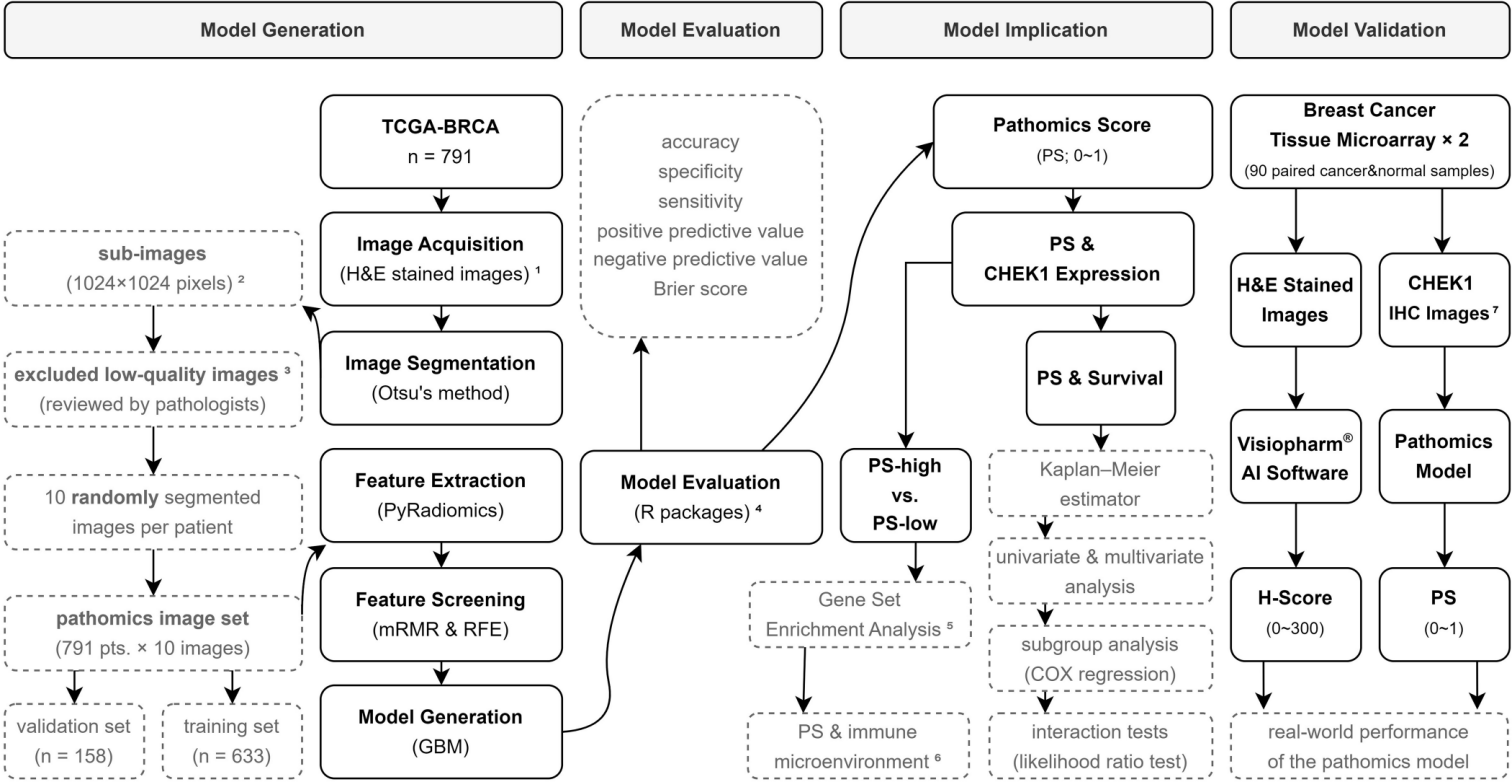
The workflow for generating, evaluating, exploring the implication, and validating the CHEK1 prediction pathomics model. 1) H&E-stained images in svs format, with a maximum magnification of 20 × or 40 × , are used. 2) 40 × images are divided into 1024 × 1024 sub-images, while 20 × images are magnified to 1024 × 1024 from 512 × 512 sub-images. 3) Images are manually reviewed by two independent pathologists. Those with obvious contamination, blurring, or blank areas exceeding 50% are defined as low quality. 4) The performance of the pathomics model is assessed using the R packages “pROC”, “ResourceSelection”, “rms”, and “rmda”. 5) The pre-defined Hallmark gene sets, and the KEGG gene sets are used for analysis. 6) Including immune cell infiltration, immune gene expression, and potential response to anti-PD-1 and anti-CTLA4 treatments are explored. 7) IHC is the abbreviation for immunohistochemistry.

**Fig 4:**
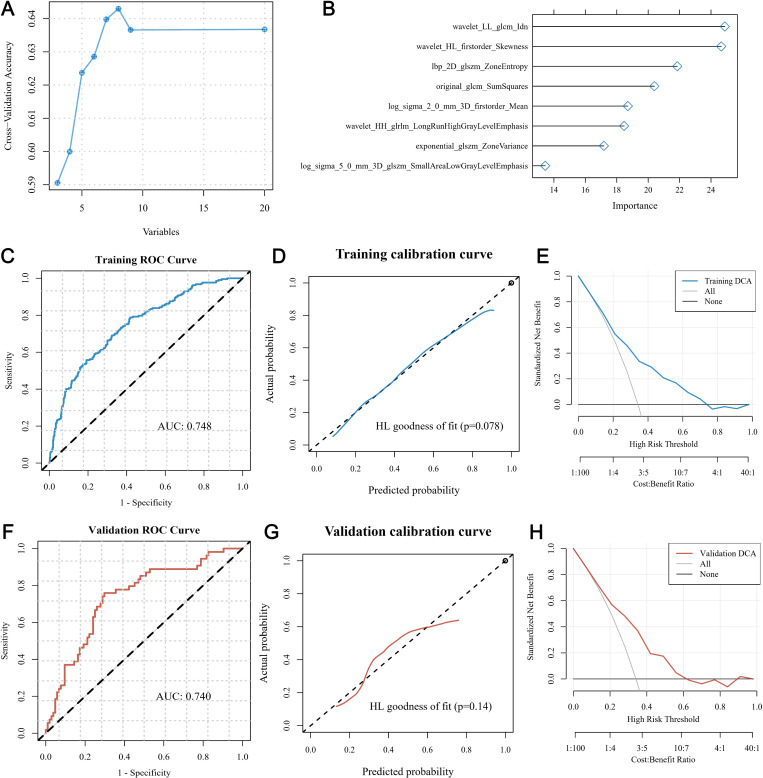
Performance evaluation of the CHEK1 prediction pathomics model. A) The top 20 features from analyzed H&E-stained images are selected using the mRMR algorithm. B) The RFE algorithm further identifies the 8 most important features. The graph shows the ROC curves of our pathomics model in the C) training and F) validation sets. The graph shows the calibration curve of our pathomics model in the D) training and G) validation sets, along with the DCA curves of our pathomics model in the E) training and H) validation sets.

Fig 3 illustrates the workflow involved in generating the pathomics model for predicting CHEK1 expression using the H&E-stained images. H&E images meeting the necessary qualifications were obtained from 791 patients from the TCGA-BRCA dataset. After a series of pre-processing steps, the randomly selected images (n = 10) for each patient resulted in a total of 7,910 segmented sub-images for all patients. These segmented sub-images were divided into the training (10 × 633 images) and validation sets (10 × 158 images) in a 4:1 ratio. The baseline characteristics of these classified sets were comparable across all subgroups (S1 Table). Furthermore, 1,488 features were extracted from the selected images in the training set, including 93 original features (both first and second order), as well as high-order features, such as Wavelet (LL, LH, HL, and HH), LoG (kernel sizes 1–5), Square, SquareRoot, Logarithm, Exponential, Gradient, and LBP2D. The top 20 extracted features of these chosen images were selected using the mRMR algorithm. Further, the RFE algorithm identified the 8 most important features (Fig 4A and 4B). The GBM algorithm was used to model the selected pathological features for predicting a gene expression.

Together, the designed pathomics model demonstrated excellent predictive performance based on the receiver operating characteristic (ROC) curves. The area under the curve (AUC) values of the model were 0.748 in the training set and 0.740 in the validation set (Fig 4C and 4F). The Hosmer-Lemeshow goodness-of-fit test demonstrated that this pathomics model exhibited exceptional consistency between the predicted probability of high gene expression and the actual expression values. The P-values of the training and validation sets were 0.078 and 0.140, respectively (Figs 4D and 4G). As illustrated in Figs 4E and 4H, the DCA values further demonstrated that this model possessed high clinical utility.

In our analysis of the relationship between the Pathomics score and CHEK1 expression levels within the dataset, we computed the Pearson correlation coefficient. The results indicated a Pearson correlation coefficient of 0.4605, suggesting a moderate positive correlation between the two variables (S2 Table) (95% confidence interval: 0.4037 to 0.5137). The correlation test yielded a t-value of 14.571 with 789 degrees of freedom and a p-value less than 2.2e-16, indicating that the correlation is statistically highly significant. We further analyzed this relationship by constructing a linear regression model, where CHEK1 expression level was the dependent variable and the Pathomics score was the independent variable. The regression analysis revealed that the estimated regression coefficient for the Pathomics score was 2.4475 (S2 Table) (standard error = 0.1680, t-value = 14.57, p-value < 2e-16). This indicates that for every unit increase in the Pathomics score, the CHEK1 expression level increases by approximately 2.4475 units on average. The intercept was estimated at 1.1988 (S2 Table) (standard error = 0.0619, t-value = 19.37, p-value < 2e-16). An analysis of the residuals showed a minimum value of -1.8271, a first quartile of -0.4724, a median of -0.0745, a third quartile of 0.4290, and a maximum value of 2.8106. The residual standard error was 0.6811, with 789 degrees of freedom. The model’s R-squared value was 0.212, and the adjusted R-squared value was 0.211(S2 Table), indicating that the Pathomics score accounts for 21.2% of the variability in CHEK1 expression levels. The F-statistic for the model was 212.3 with 1 and 789 degrees of freedom, and a p-value less than 2.2e-16, demonstrating that the model is overall statistically significant. These findings indicate that the Pathomics score is a significant positive predictor of CHEK1 expression levels. Both the correlation and regression analyses indicate that this relationship is statistically highly significant.

### High PS value is associated with poor prognostic outcomes

**Fig 5:**
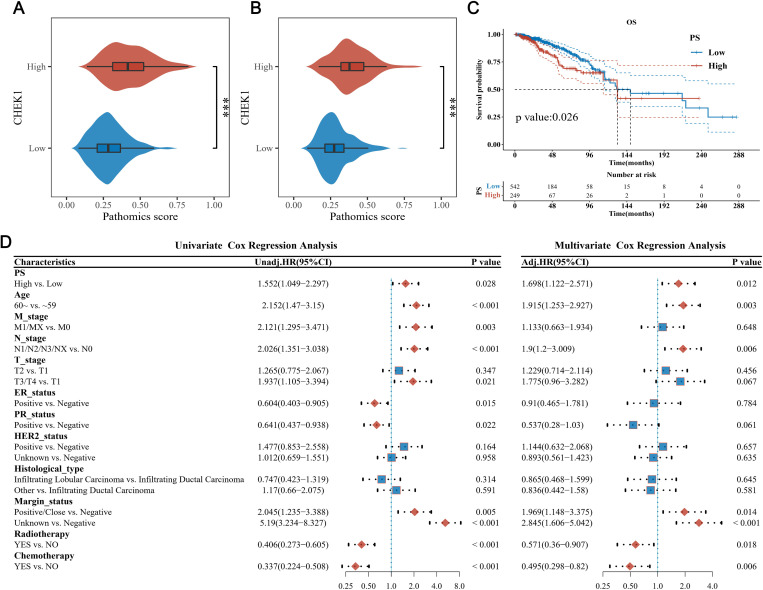
Evaluation of the prognostic significance of PS values in BrC using the TCGA-BRCA dataset. The correlation between PS and CHEK1 expression levels in the A) training and B) validation sets are assessed along with the statistical differences using the Wilcoxon test. C) The graph shows the KM curves for OS in patients with high and low PS. The median OS for the “PS-high” and “PS-low” groups are indicated in red and blue text, respectively. D) The univariate and multivariate Cox regression analyses of potential risk factors for OS are presented. An HR value greater than 1 indicates a risk factor, while an HR value less than 1 indicates a protective factor. Abbreviations: *, P < 0.001; m, months; OS, overall survival; HR, hazard ratio.

In this study, the designed pathomics model successfully predicted the expression levels of CHEK1 on a specific H&E-stained image. These expression levels were represented as the PS values, ranging from 0 to 1. PS values were estimated for the selected 791 patients with qualified H&E-stained images to evaluate the performance of PS in predicting the prognosis of BrC. Using the R package “survminer”, a cut-off value of 0.3852 was determined to distinguish between the “PS-high” and “PS-low” groups. Accordingly, a total of 249 patients were classified as “PS-high”, and the remaining 542 patients were denoted as “PS-low”. The baseline characteristics of both the classified groups were similar (P > 0.05 in all subgroups, Table 2). As depicted in Figs 5A and 5B, high PS value corresponded to high expression levels of CHEK1 in both the training and validation sets, respectively. Furthermore, significant differences in CHEK1 expression levels were observed between the PS-high and PS-low groups in both the classified sets (Wilcoxon test, P < 0.001). Although the overall prediction accuracy for BrC subtypes was 71.1% (S7 Fig), the accuracy rates for each subtype were as follows: 78% for Luminal A, 57% for Luminal B, 69% for Basal, 59% for HER2, and 73% for others.

**Table 2:** Baseline Characteristics of Patients in the PS-high and PS-low Groups.

Variables	Total (n = 791)	PS-Low (n = 542)	PS-High (n = 249)	p
**Age, n (%)**				
<60	439 (55)	305 (56)	134 (54)	0.569
≥60	352 (45)	237 (44)	115 (46)	
**T Stage, n (%)**				
T1	192 (24)	145 (27)	47 (19)	< 0.001
T2	466 (59)	295 (54)	171 (69)	
T3/T4	133 (17)	102 (19)	31 (12)	
**N Stage, n (%)**				
N0	365 (46)	250 (46)	115 (46)	1
N1/N2/N3/NX	426 (54)	292 (54)	134 (54)	
**M Stage, n (%)**				
M0	665 (84)	457 (84)	208 (84)	0.861
M1/MX	126 (16)	85 (16)	41 (16)	
**ER Status, n (%)**				
Negative	182 (23)	93 (17)	89 (36)	< 0.001
Positive	609 (77)	449 (83)	160 (64)	
**PR Status, n (%)**				
Negative	259 (33)	140 (26)	119 (48)	< 0.001
Positive	532 (67)	402 (74)	130 (52)	
**HER2 Status, n (%)**				
Negative	415 (52)	282 (52)	133 (53)	0.358
Positive	133 (17)	86 (16)	47 (19)	
Unknown	243 (31)	174 (32)	69 (28)	
**Histological Type, n (%)**				
Infiltrating Ductal Carcinoma	574 (73)	372 (69)	202 (81)	< 0.001
Infiltrating Lobular Carcinoma	143 (18)	120 (22)	23 (9)	
Other	74 (9)	50 (9)	24 (10)	
**Margin Status, n (%)**				
Negative	665 (84)	449 (83)	216 (87)	0.365
Positive/Close	79 (10)	59 (11)	20 (8)	
Unknown	47 (6)	34 (6)	13 (5)	
**Chemotherapy, n (%)**				
No	332 (42)	236 (44)	96 (39)	0.214
Yes	459 (58)	306 (56)	153 (61)	
**Radiotherapy, n (%)**				
No	381 (48)	257 (47)	124 (50)	0.585
Yes	410 (52)	285 (53)	125 (50)	

The median OS rate for patients with high PS was 131.97 months, while it was 148.53 months for patients with low PS (P = 0.026; Fig 5C). The univariate and multivariate analyses showed that high PS could be a risky factor influencing the OS rate of patients. The HR values were 1.552 (95%CI: 1.049–2.297, P = 0.028) and 1.698 (95%CI: 1.122–2.571, P = 0.012) in the univariate and multivariate analyses, respectively (Fig 5D). The subgroup analysis and interaction tests showed that the risk effect of high PS scores was independent of most included clinical characteristics (P > 0.05; S8 Fig), except for PR status (P = 0.026) and HER2 status (P = 0.021 for positive *vs.* negative and P = 0.158 for unknown vs. negative).

### Potential implications of high PS

**Fig 6:**
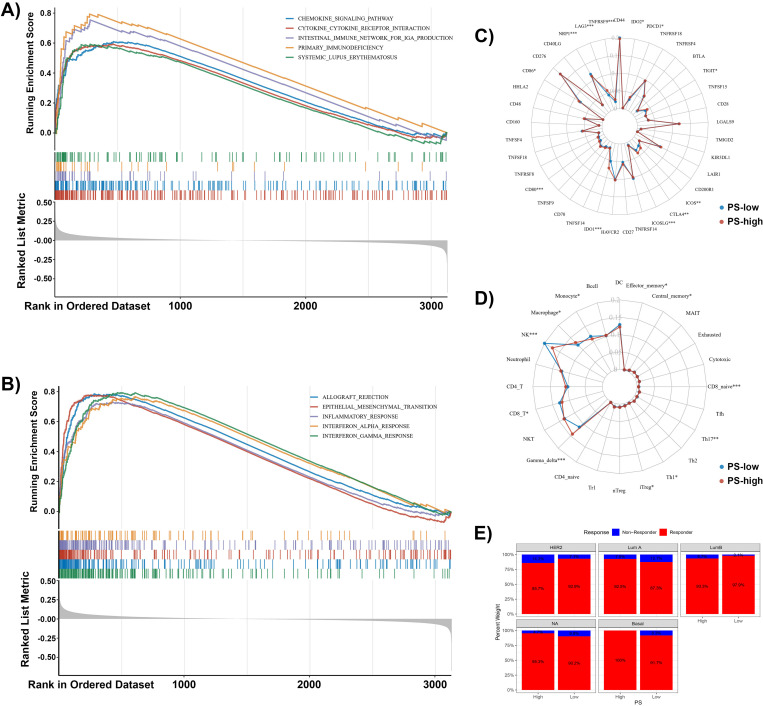
Potential implications of high PS in BrC. The image shows the enriched pathways using A) the KEGG gene set and B) the Hallmark gene set from Gene Set Enrichment Analysis (GSEA). C) The graph shows the differences in the immune gene expression between the PS-high and PS-low groups. D) The data show the differences in immune cell infiltration between the PS-high and PS-low groups. E) The TIDE scores predict the differences in response to anti-PD-1 and anti-CTLA4 treatments between the PS-high and PS-low groups.

Further, the potential implications of high PS were explored by comparing the differences in the expression of pre-defined gene sets between the PS-high and PS-low groups using the GSEA strategy. The enriched pathways for high PS using KEGG gene sets included 1) chemokine signalling pathway, 2) cytokine receptor interaction, 3) intestinal immune network for IgA production, 4) primary immunodeficiency, and 5) systemic lupus erythematosus (Fig 6A). Similarly, the top 5 enriched pathways for high PS using Hallmark gene sets included 1) allograft rejection, 2) epithelial-mesenchymal-transition, 3) inflammatory responses, 4) interferon-alpha responses, and 5) interferon-gamma responses (Fig 6B).

As the notified enriched pathways were mostly immune-related, the relationship between high PS and cancer immune microenvironments was further investigated. It was observed from the assessments of the Wilcoxon rank sum test that the expression levels of the specific immune-related genes were significantly higher in the PS-high group compared to the PS-low group (Fig 6C; S3 Table), such as TNFRSF9 (P < 0.001), LAG3 (P < 0.001), CD80 (P < 0.001), IDO1 (P < 0.001), ICOSLG (P < 0.001), CTLA4 (P < 0.01), ICOS (P < 0.01), CD86 (P < 0.05), TIGIT (P < 0.05), PDCD1 (P < 0.05), and IDO2 (P < 0.05). Several immune cells showed significant changes in the PS-high patients (Fig 6D; S4 Table), indicating upregulated gamma delta (P < 0.001), macrophages (P < 0.05), iTregs (P < 0.05), Th1 (P < 0.05), central memory (P < 0.05), and effector memory cells (P < 0.05), as well as down-regulated natural killer (NK, P < 0.001), CD8 naive (P < 0.001), Th17 (P < 0.01), monocytes (P < 0.05), and CD8 T (P < 0.05). Besides,TIDE predictions indicated potential differences in immunotherapy response among patients with varying PS values across different breast cancer subtypes. The results showed that patients with high and low PS values exhibited different predicted response rates to immunotherapy in Basal, HER2, Luminal A, Luminal B, and NA breast cancer subtypes (Fig 6E). Specifically, the percentages of predicted responders in the high PS group compared to the low PS group were as follows: HER2 (85.7% vs. 92.9%), Luminal A (87.3% vs. 92.5%), Luminal B (93.3% vs. 97.9%), NA (95.3% vs. 90.2%), and Basal (100% vs. 91.7%). To assess whether the pathomic model of CHEK1 expression could guide patient immunotherapy, we analyzed the correlation between tumor mutational burden (TMB) and CHEK1 expression levels. In the Luminal A subtype (R = 0.25, P = 6.4e-06) and Luminal B subtype (R = 0.20, P = 0.039), a significant positive correlation was observed between CHEK1 expression and TMB. Similarly, a significant positive correlation was found in the NA subtype (R = 0.26, P = 2.1e-07). However, for HER2 (R = 0.22, P = 0.21) and Basal (R = 0.24, P = 0.061) subtypes, the correlation did not reach statistical significance (S9 Fig). These findings suggest that higher CHEK1 expression may be associated with increased TMB in certain breast cancer subtypes, potentially influencing the effectiveness of immunotherapy.

### Real-world performance of this pathomics model

**Fig 7:**
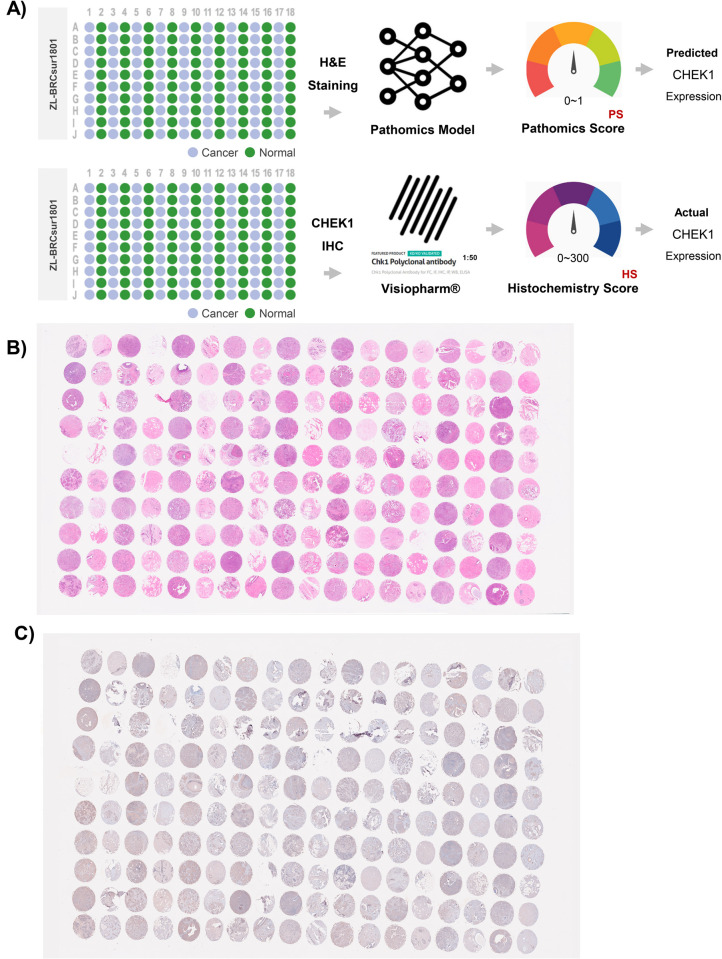
Validation of the CHEK1 prediction pathomics model using BrC TMA. A) The workflow validates the real-world performance of this CHEK1 prediction pathomics model using BrC TMA slides.B) The data show the H&E-stained image of the BrC in TMA.C) The IHC image of the BrC TMA shows the stained CHEK1 antibodies.

**Fig 8:**
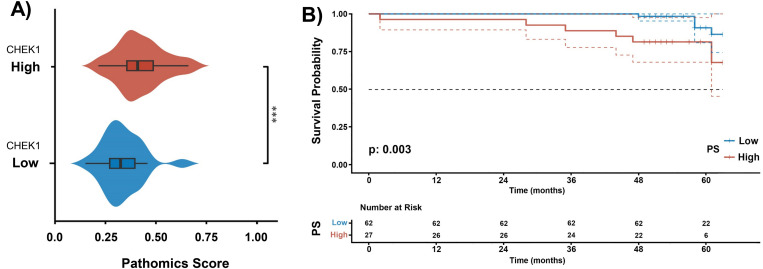
Correlation and survival analysis of CHEK1 expression in BrC TMA. A) The image shows the correlation between predicted PS and actual CHEK1 expression levels (Histochemistry Score, HS) in TMA, where statistical differences are assessed using the Wilcoxon test. **E)** The data from TMA present the KM curves for OS in patients with high and low PS.

Eventually, two sets of BrC TMA slides were applied to demonstrate the real-world performance of the CHEK1 prediction pathomics model. The scanned images from the H&E-stained slides were exported to the pathomics model to obtain corresponding PS values (0–1) for predicting CHEK1 expression. Meanwhile, the definite levels of CHEK1 expression were assessed by calculating the HS values (0–300) from IHC staining images using CHEK1 antibodies (Figs 7A–C). By applying the R package “survminer”, a threshold of 141.41 was established to differentiate the “HS-high” and “HS-low” groups. Consequently, patients (n = 50) were categorized as “HS-high”, while the remaining patients (n = 39) were categorized as “HS-low”. The baseline characteristics of both groups exhibited similarity (S5 Table). The PS levels were significantly higher in samples with high HS values compared to samples with low HS values (P < 0.001; Fig 8A).

Furthermore, patients with high PS values had worse survival outcomes than those with low PS values (Fig 8B). After a 60-month follow-up, the median OS was 64 months in the high PS group of patients, while it failed to reach in the low PS group. Similar to the findings from the TCGA-BRCA dataset, the univariate and multivariate Cox regression analyses indicated that the PS prediction based on TMA data suggested that high CHEK1 expression could be a risk factor for the OS rate of patients (S10 Fig). The AUC value of the pathomics model was 0.728 for H&E-stained images scanned from TMA slides. The reliability and clinical utility attributes of the model were further confirmed by the Hosmer-Lemeshow goodness-of-fit test and DCA, respectively (S11 Fig). However, the overall prediction accuracy was reportedly 92.1% for all BrC subtypes (S12 Fig). The specific types indicated were as follows: 92% for HR + /HER2 + cases, 92% for HR + /HER2- cases, 96% for HR-/HER2 + cases, and 80% for HR-/HER2- cases. The detailed HS, PS, and clinical data associated with TMA are available in S6 Table.

## Discussion

Firstly, the significance of CHEK1 expression in the prognosis of BrC was investigated, indicating that its overexpression could be a risk factor for OS, especially in the early stage, using 96 months post-diagnosis as the landmark. Secondly, a pathomics model was developed that could accurately predict the CHEK1 expression using the H&E-stained images and demonstrate its performance efficacy. Thirdly, the reliability of the predicted CHEK1 expression levels (PS values) was validated in predicting the BrC prognosis. A high PS value could correspond to a high CHEK1 expression level in both the validation image set and the real-world TMA slides. Finally, the potential molecular implications of high PS were investigated, in which high PS was typically associated with changes in the immune microenvironment towards offering better responses to anti-PD-1 and anti-CTLA4 treatments.

This study demonstrates the improved management of BrC for various reasons. First, predicting the expression level of a specific gene using a generated pathomics model is much more cost-effective in terms of time and resources compared to the traditional gene expression measurement methods, such as real-time quantitative polymerase chain reaction (qRT-PCR), Western Blot, enzyme-linked immunosorbent assay (ELISA), IHC, and immunofluorescence strategies. The H&E-stained images serve as the foundation for making a pathological diagnosis, eliminating the need for extra sample collection and processing procedures. These attributes can increase the utilization of predicted CHEK1 expression level as a means of predicting the BrC prognosis. Accordingly, our findings suggested the importance of CHEK1 expression in guiding the immunotherapy for BrC treatment. To the best of our knowledge, this report on the CHEK1 prediction pathomics model, for the first time, indicated the promotion of personalized risk stratification and individualized precision therapy in BrC. Moreover, it should be noted that it was quite challenging to compare our findings to previous publications as no reports were evident. For instance, no publications were found on PubMed using the keywords “pathomics”, “CHEK1”, and “breast cancer”. Nevertheless, two partial findings could be compared: 1) the prognostic significance of CHEK1 in cancer and 2) the reliability and performance of pathomics models.

As specified in the introduction, CHEK1 overexpression was frequently observed in solid tumors, indicating its role in the prognosis of melanoma, lung adenocarcinoma, hepatocellular carcinoma, bladder cancer, brain cancer, ovarian cancer, and BrC [17–21]. Accordingly, our findings in this study were consistent with the reported literature. Nevertheless, some exceptions have been reported, in which high CHEK1 expression could also be associated with better prognostic outcomes in colorectal and gastric cancers [17]. Previous studies demonstrated the reliability and performance of pathomics models in various cancers, including colorectal cancer [28,59], gastric cancer [29,60], bladder cancer [61,62], hepatocellular carcinoma [63], clear cell renal cell carcinoma [64], rectal cancer [65], gliomas [66], non-small cell lung cancer [67], and BrC [68]. These pathomics models could be used as a single model or as a part of a comprehensive model that might involve radiomics, genomics, transcriptomics, proteinomics, and other omics techniques [24]. Typically, the pathomics models generated with machine learning-based methods can be reliable in predicting cancer prognosis [29,61,62,64,66], subtype classification [67,68], recurrence status [60,63], treatment response [65], postoperative outcomes [59], as well as guiding the treatment of metastatic diseases [28]. Together, these results emphasized the significance of pathomics models in improving cancer management in clinical practice.

Despite the success, our study possesses several limitations that should be considered for further improvement. First, our analysis was limited to patients from the TCGA-BRCA dataset, which might not be representative of all BrC patients. Previous studies have extensively explored the role of CHEK1 in TNBC, particularly in regulating replication stress and chemotherapy resistance [69]. Recent evidence, however, suggests that CHEK1 also plays a critical role in hormone receptor-positive (HR+) breast cancer subtypes, particularly LumA and LumB. Research has shown that CHEK1 maintains genomic stability via the ATR-CHK1 signaling pathway, a mechanism conserved across all breast cancer subtypes [69]. In Luminal subtypes, overexpression of CHEK1 promotes tumor cell survival by inhibiting replication stress-induced apoptosis [70]. Our study found that high CHEK1 expression is significantly associated with poorer survival outcomes in the LumA/LumB subtypes, consistent with results from independent cohorts such as METABRIC [70]. Clinical studies have demonstrated that the CHEK1 inhibitor AZD7762 synergizes with CDK4/6 inhibitors in HR+ breast cancer models, highlighting the potential of CHEK1 as a cross-subtype therapeutic target [71]. Our pathological model uniquely emphasizes the independent prognostic value of CHEK1 in non-TNBC subtypes, particularly LumA/LumB, suggesting that CHEK1 may influence the immune microenvironment by regulating genomic instability and T cell infiltration. Subtype stratification analysis revealed a significant correlation between tumor mutational burden (TMB) and CHEK1 expression in LumA/LumB, but not in TNBC. TNBC typically exhibits higher baseline TMB and genomic instability [72], which may obscure the individual regulatory effect of CHEK1 on TMB. In contrast, the lower TMB in Luminal subtypes makes CHEK1-driven replication stress a major contributor to TMB accumulation [69]. In Luminal subtypes, CHEK1 overexpression may suppress the STING pathway, thereby reducing type I interferon secretion, which lowers antigen presentation efficiency and promotes immune escape [73]. Immune infiltration in TNBC may mitigate this effect. Targeting CHEK1 could reduce genomic instability in LumA/LumB subtypes and improve responses to immunotherapy. In TNBC, however, homologous recombination defects associated with BRCA1/2 mutations may dominate the regulation of TMB [74]. While our pathomics model demonstrated good predictive performance, it had not been validated in real-world cases. Considering these attributes, further studies with a larger sample size will be needed to demonstrate the clinical utility of this pathomics model, as well as the potential benefits of CHEK1-targeted therapy in BrC.

## Conclusion

In summary, the study presented that high CHEK1 expression levels in BrC typically could indicate poor prognostic outcomes. Our novel pathomics model could accurately predict CHEK1 expression by analyzing H&E stained images using machine learning algorithms. Furthermore, the PS values could be used to predict prognosis and guide immunotherapy for BrC patients.

## Supporting information

S1 Fig Preprocessing and segmentation of H&E-stained breast tumor tissue images. H&E-stained images of formalin-fixed, paraffin-embedded breast tumor tissues from the TCGA database. Images are divided using the OTSU algorithm to distinguish tissue areas from the background. Sub-images of 1024 × 1024 pixels are extracted for further analysis.
(JPG)

S2 Fig CHEK1 expression levels in various cancer types. Box plot of CHEK1 expression (log2(TPM + 1)) in normal and tumor tissues across 34 cancer types using RNA-seq data from the UCSC Xena browser.
(JPG)

S3 Fig CHEK1 expression in breast cancer subtypes. CHEK1 expression levels in various breast cancer subtypes including HER2-positive (HER2), Luminal A (Lum A), Luminal B (Lum B), and triple-negative (Basal) compared to normal controls (CN).
(JPG)

S4 Fig Impact of CHEK1 expression on breast cancer overall survival (OS). Kaplan-Meier survival curves showing the correlation between CHEK1 expression levels and overall survival (OS) in breast cancer patients.
(JPG)

S5 Fig Landmark analysis of overall survival based on CHEK1 expression in breast cancer. Kaplan-Meier survival curves depicting overall survival (OS) of breast cancer patients stratified by CHEK1 expression levels at various time points post-diagnosis (24-month intervals).
(JPG)

S6 Fig Subgroup analysis and interaction tests of CHEK1 overexpression on overall survival in breast cancer. Forest plot showing the hazard ratios (HRs) and 95% confidence intervals (CIs) for the effect of CHEK1 overexpression on overall survival (OS) across different baseline characteristics in breast cancer patients.
(JPG)

S7 Fig Prediction accuracy of breast cancer subtypes based on CHEK1 expression. Prediction accuracy for breast cancer subtypes using a pathomics model incorporating CHEK1 expression.
(JPG)

S8 Fig Subgroup analysis and interaction tests of prognostic scores on overall survival in breast cancer. Forest plot showing the hazard ratios (HRs) and 95% confidence intervals (CIs) for the interaction of risk effect from high prognostic scores (PS) with various clinical characteristics in breast cancer patients.
(JPG)

S9 Fig Correlation between CHEK1 expression and tumor mutation burden (TMB) in breast cancer subtypes. Scatter plots showing the correlation between CHEK1 expression and tumor mutation burden (TMB) across different breast cancer subtypes.
(TIF)

S10 Fig Univariate and multivariate Cox regression analyses of CHEK1 expression on overall survival. Forest plot showing univariate and multivariate Cox regression analyses for the overall survival (OS) of breast cancer patients based on prognostic scores (PS) and various clinical characteristics.
(JPG)

S11 Fig Performance and validation of the pathomics model for predicting breast cancer prognosis. ROC curve, calibration curve, and decision curve analysis (DCA) for the pathomics model based on CHEK1 expression and H&E-stained images from TMA slides.
(JPG)

S12 Fig Prediction accuracy for breast cancer subtypes based on CHEK1 expression. Prediction accuracy of the pathomics model for various breast cancer (BrC) subtypes.
(JPG)

S1 Table Baseline characteristics of patients in the train and validation groups. (DOCX)

S2 Table Supplementary 6. (DOCX)

S3 Table Immune gene expression differences between the PS-high and PS-low groups. (DOCX)

S4 Table Immune cell infiltration differences between the PS-high and PS-low groups (DOCX)

S5 Table Baseline characteristics of patients in the PS-low and PS-high groups using the BrC. Tissue Microarray.
(DOCX)

S6 Table H-score, PS, and clinical data for breast cancer tissue microarray. (XLSX)
