# Supplementary figures and images for: Predicting breast cancer prognosis based on a novel pathomics model through CHEK1 expression analysis using machine learning algorithms
Source: PLoS One. 2025 May 9;20(5):e0321717. doi: 10.1371/journal.pone.0321717 (PMC12064205; doi:10.1371/journal.pone.0321717)

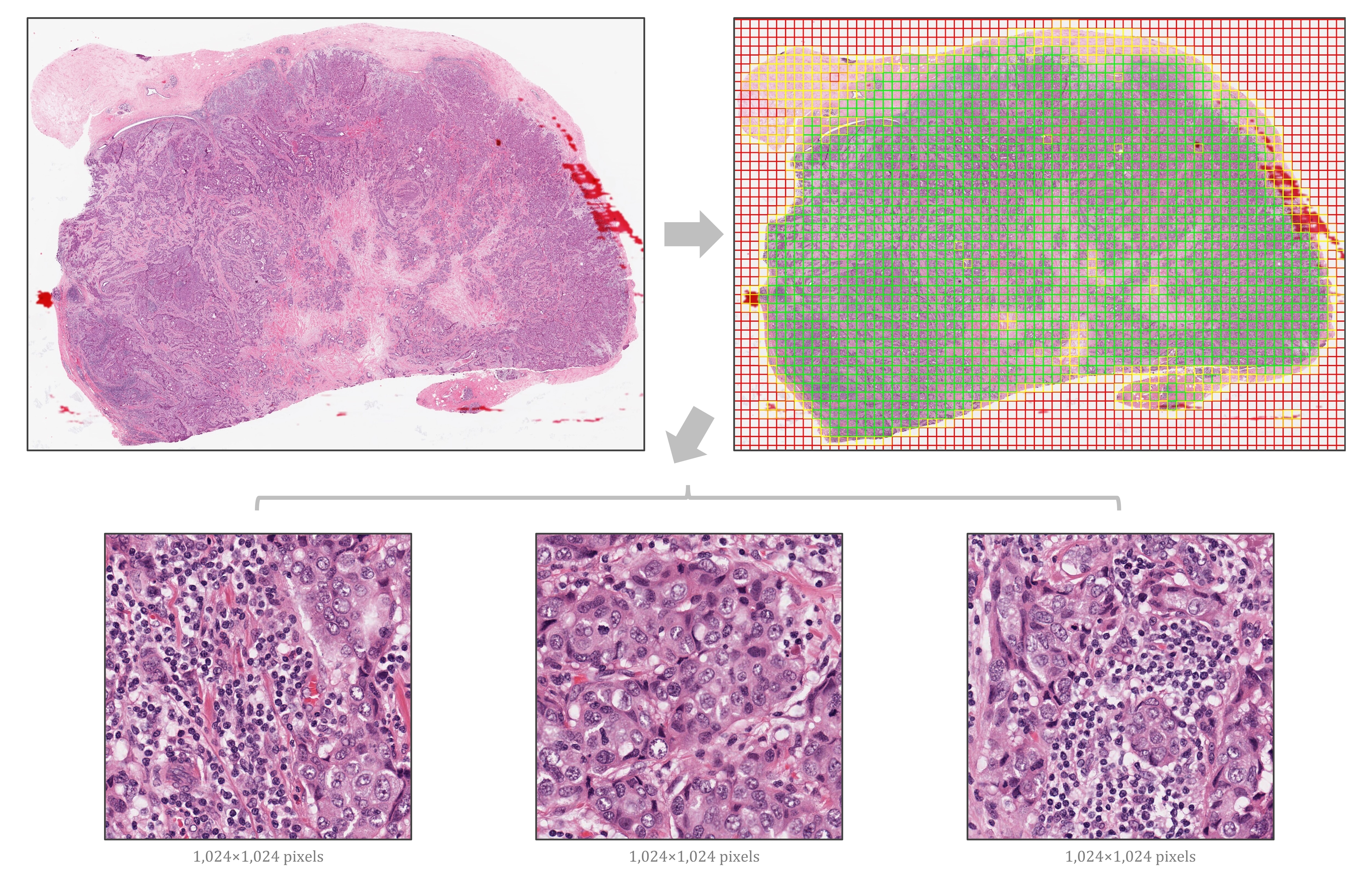

Supplement: S1 Fig — H&E-stained images of formalin-fixed, paraffin-embedded breast tumor tissues from the TCGA database. Images are divided using the OTSU algorithm to distinguish tissue areas from the background. Sub-images of 1024 × 1024 pixels are extracted for further analysis. (JPG) [file pone.0321717.s001.jpg]

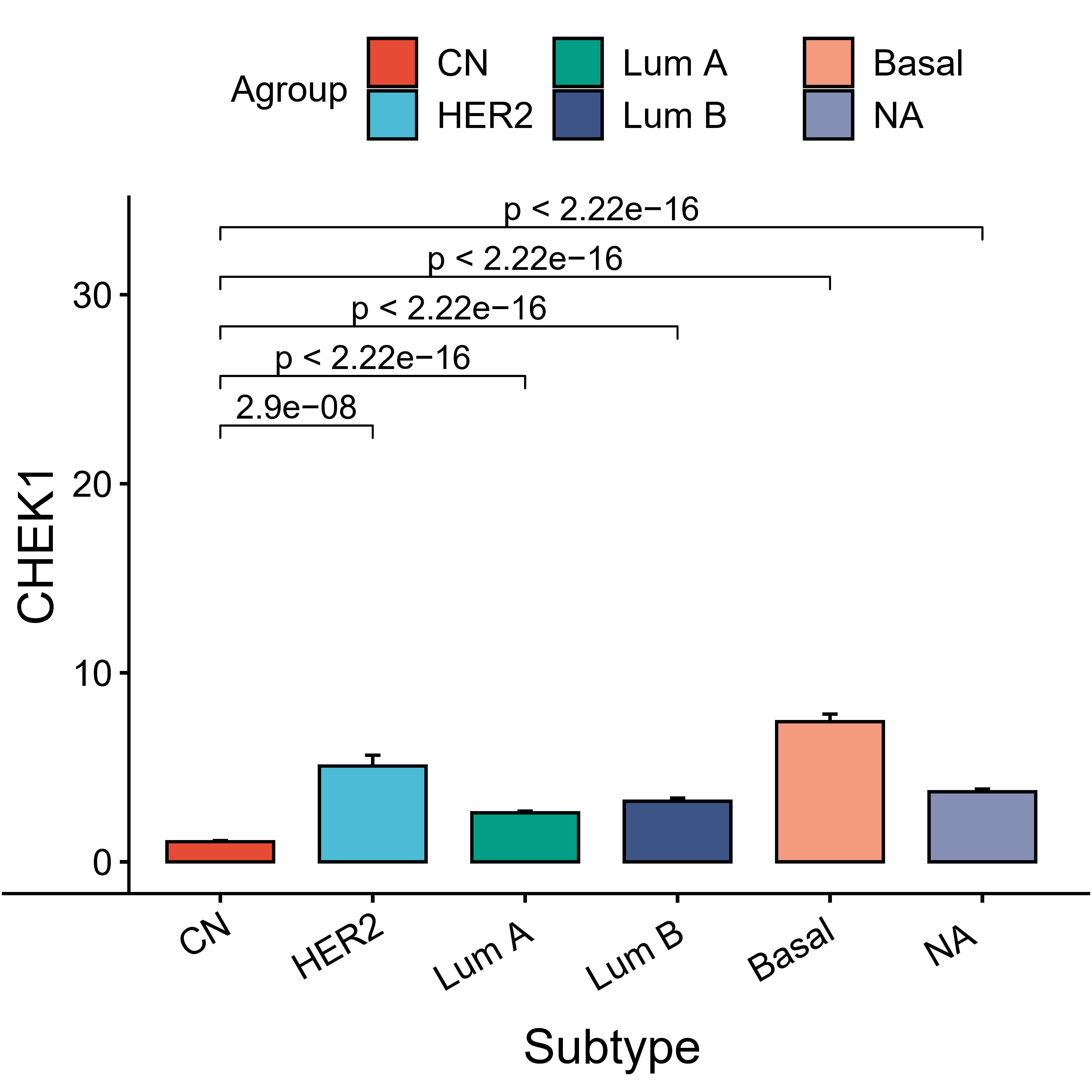

Supplement: S3 Fig — CHEK1 expression levels in various breast cancer subtypes including HER2-positive (HER2), Luminal A (Lum A), Luminal B (Lum B), and triple-negative (Basal) compared to normal controls (CN). (JPG) [file pone.0321717.s003.jpg]

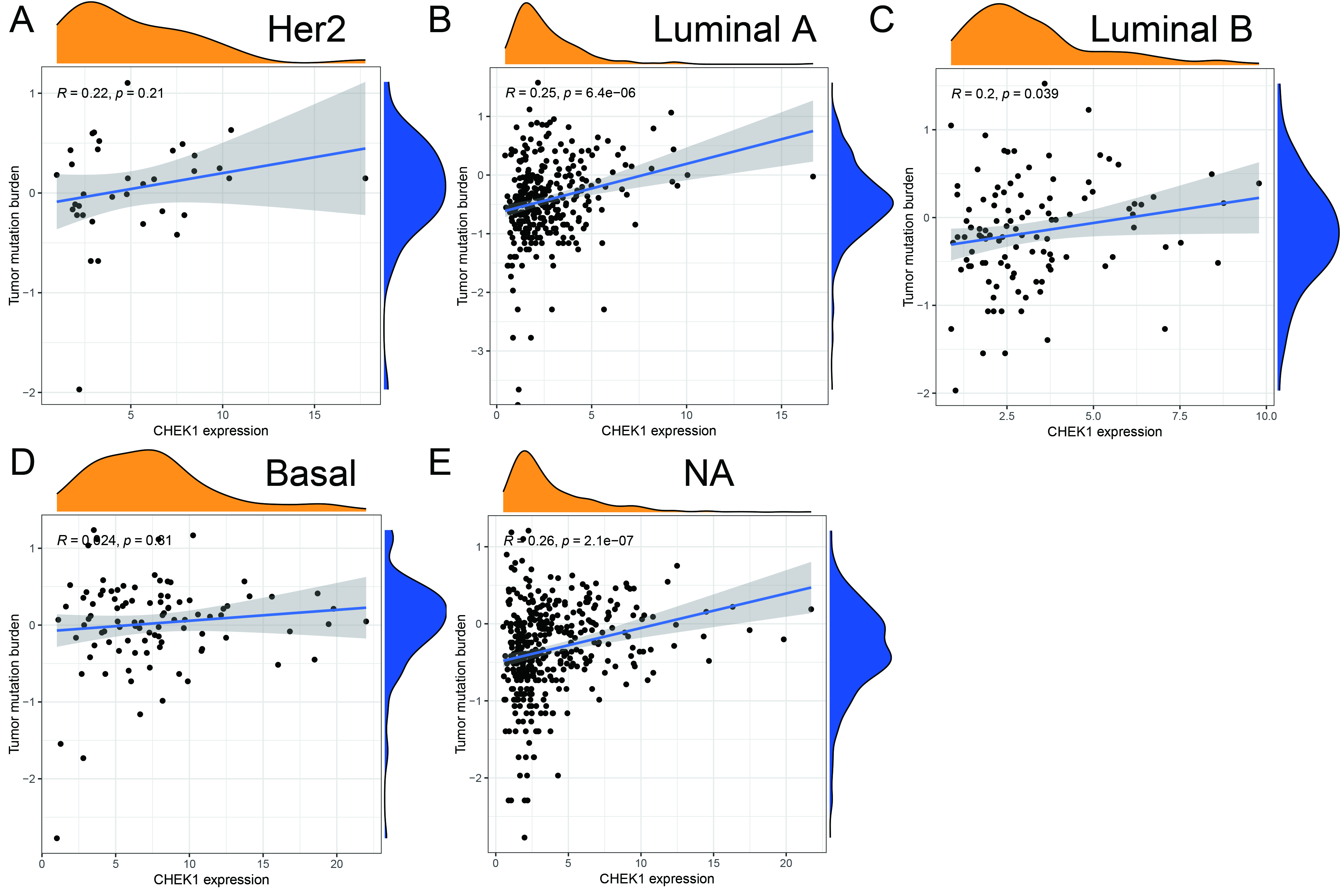

Supplement: S9 Fig — Scatter plots showing the correlation between CHEK1 expression and tumor mutation burden (TMB) across different breast cancer subtypes. (TIF) [file pone.0321717.s009.tif]
