# Supplementary material for: Predicting breast cancer prognosis based on a novel pathomics model through CHEK1 expression analysis using machine learning algorithms
Source: PLoS One. 2025 May 9;20(5):e0321717. doi: 10.1371/journal.pone.0321717 (PMC12064205; doi:10.1371/journal.pone.0321717)
Supplement: S1 Table — (DOCX) [file pone.0321717.s013.docx]

**Baseline Characteristics of Patients in the Train and Validation Groups**

| **Variables** | **Total  (n = 791)** | **Train  (n = 633)** | **Validation  (n = 158)** | **p** |  |
| --- | --- | --- | --- | --- | --- |
| **CHEK1, n (%)** | | | | | |
| Low | 520 (66) | 416 (66) | 104 (66) | 1 |  |
| High | 271 (34) | 217 (34) | 54 (34) |  |  |
| **Age, n (%)** | | | | | |
| ＜60 | 439 (55) | 343 (54) | 96 (61) | 0.162 |  |
| ≥60 | 352 (45) | 290 (46) | 62 (39) |  |  |
| **T Stage, n (%)** | | | | | |
| T1 | 192 (24) | 151 (24) | 41 (26) | 0.533 |  |
| T2 | 466 (59) | 371 (59) | 95 (60) |  |  |
| T3/T4 | 133 (17) | 111 (18) | 22 (14) |  |  |
| **N Stage, n (%)** | | | | | |
| N0 | 365 (46) | 289 (46) | 76 (48) | 0.644 |  |
| N1/N2/N3/NX | 426 (54) | 344 (54) | 82 (52) |  |  |
| **M Stage, n (%)** | | | | | |
| M0 | 665 (84) | 534 (84) | 131 (83) | 0.746 |  |
| M1/MX | 126 (16) | 99 (16) | 27 (17) |  |  |
| **ER Status, n (%)** | | | | | |
| Negative | 182 (23) | 141 (22) | 41 (26) | 0.381 |  |
| Positive | 609 (77) | 492 (78) | 117 (74) |  |  |
| **PR Status, n (%)** | | | | | |
| Negative | 259 (33) | 206 (33) | 53 (34) | 0.885 |  |
| Positive | 532 (67) | 427 (67) | 105 (66) |  |  |
| **HER2 Status, n (%)** | | | | | |
| Negative | 415 (52) | 328 (52) | 87 (55) | 0.442 |  |
| Positive | 133 (17) | 104 (16) | 29 (18) |  |  |
| Unknown | 243 (31) | 201 (32) | 42 (27) |  |  |
| **Histological Type, n (%)** | | | | | |
| Infiltrating Ductal Carcinoma | 574 (73) | 458 (72) | 116 (73) | 0.475 |  |
| Infiltrating Lobular Carcinoma | 143 (18) | 112 (18) | 31 (20) |  |  |
| Other | 74 (9) | 63 (10) | 11 (7) |  |  |
| **Margin Status, n (%)** | | | | | |
| Negative | 665 (84) | 535 (85) | 130 (82) | 0.61 |  |
| Positive/Close | 79 (10) | 63 (10) | 16 (10) |  |  |
| Unknown | 47 (6) | 35 (6) | 12 (8) |  |  |
| **Radiotherapy, n (%)** | | | | | |
| No | 381 (48) | 309 (49) | 72 (46) | 0.521 |  |
| Yes | 410 (52) | 324 (51) | 86 (54) |  |  |
| **Chemotherapy, n (%)** | | | | | |
| No | 332 (42) | 265 (42) | 67 (42) | 0.974 |  |
| Yes | 459 (58) | 368 (58) | 91 (58) |  |  |
| **OS** | | | | | |
| Alive, n (%) | 682 (86) | 543 (86) | 139 (88) | 0.558 |  |
| Dead, n (%) | 109 (14) | 90 (14) | 19 (12) |  |  |
| Median OS, m (Q1, Q3) | 29.37  (16.17, 57.83) | 29.3  (16.27, 56.4) | 30.63  (16.05, 61.72) | 0.851 |  |
