# Supplementary material for: Predicting breast cancer prognosis based on a novel pathomics model through CHEK1 expression analysis using machine learning algorithms
Source: PLoS One. 2025 May 9;20(5):e0321717. doi: 10.1371/journal.pone.0321717 (PMC12064205; doi:10.1371/journal.pone.0321717)
Supplement: S2 Table — (DOCX) [file pone.0321717.s014.docx]

| **Statistic** | **Value** | **95% CI / SE** | **t-value** | **p-value** |
| --- | --- | --- | --- | --- |
| **Pearson Correlation** | 0.4605 | 0.4037 to 0.5137 | 14.571 | < 2.2e-16 |
| **Regression Coefficients** |  |  |  |  |
| Intercept | 1.1988 | 0.0619 | 19.37 | < 2e-16 |
| Pathomics_score | 2.4475 | 0.168 | 14.57 | < 2e-16 |
| **Residuals** |  |  |  |  |
| Minimum | -1.8271 |  |  |  |
| 1st Quartile | -0.4724 |  |  |  |
| Median | -0.0745 |  |  |  |
| 3rd Quartile | 0.429 |  |  |  |
| Maximum | 2.8106 |  |  |  |
| **Model Fit Statistics** |  |  |  |  |
| Residual Standard Error | 0.6811 |  |  |  |
| Degrees of Freedom | 789 |  |  |  |
| Multiple R-squared | 0.212 |  |  |  |
| Adjusted R-squared | 0.211 |  |  |  |
| F-statistic | 212.3 |  |  | < 2.2e-16 |

**Supplementary 6**
