# Supplementary material for: Predicting breast cancer prognosis based on a novel pathomics model through CHEK1 expression analysis using machine learning algorithms
Source: PLoS One. 2025 May 9;20(5):e0321717. doi: 10.1371/journal.pone.0321717 (PMC12064205; doi:10.1371/journal.pone.0321717)
Supplement: S3 Table — (DOCX) [file pone.0321717.s015.docx]

**Immune Gene Expression Differences Between the PS-high and PS-low Groups**

| **Gene** | **High** | **Low** | **Diff.** | **P** | **Sig.** |
| --- | --- | --- | --- | --- | --- |
| TNFRSF9 | 0.714415 | 0.543818 | up | 0.00090617 | *** |
| LAG3 | 1.538185 | 1.169515 | up | 2.46E-06 | *** |
| NRP1 | 3.014424 | 3.185207 | down | 0.000225824 | *** |
| CD80 | 0.573829 | 0.45689 | up | 6.99E-05 | *** |
| IDO1 | 1.922803 | 1.393069 | up | 0.000256122 | *** |
| ICOSLG | 0.272819 | 0.215221 | up | 0.000740114 | *** |
| CTLA4 | 1.100449 | 0.858147 | up | 0.001150812 | ** |
| ICOS | 0.938664 | 0.709831 | up | 0.001066978 | ** |
| CD86 | 2.286193 | 2.155631 | up | 0.037996882 | * |
| TIGIT | 1.103052 | 0.930743 | up | 0.014881477 | * |
| PDCD1 | 0.988217 | 0.810439 | up | 0.021483256 | * |
| IDO2 | 0.067711 | 0.063831 | up | 0.034607664 | * |
| CD44 | 5.283051 | 5.361671 | down | 0.34703336 |  |
| CD40LG | 0.755779 | 0.732871 | up | 0.920197849 |  |
| CD276 | 4.649483 | 4.656558 | down | 0.577153268 |  |
| HHLA2 | 0.025342 | 0.022953 | up | 0.147852612 |  |
| CD48 | 1.423053 | 1.350475 | up | 0.347634998 |  |
| CD160 | 0.233293 | 0.226628 | up | 0.925652851 |  |
| TNFSF4 | 1.482546 | 1.536465 | down | 0.344376769 |  |
| TNFSF18 | 0.330507 | 0.308829 | up | 0.727488707 |  |
| TNFRSF8 | 0.511479 | 0.439765 | up | 0.147013759 |  |
| TNFSF9 | 0.779426 | 0.721476 | up | 0.404105603 |  |
| CD70 | 0.618497 | 0.510574 | up | 0.079247314 |  |
| TNFSF14 | 0.486359 | 0.434834 | up | 0.118587025 |  |
| HAVCR2 | 2.749466 | 2.718251 | up | 0.561464775 |  |
| CD27 | 1.539614 | 1.384425 | up | 0.115883013 |  |
| TNFRSF14 | 2.65487 | 2.748775 | down | 0.121662856 |  |
| CD200R1 | 0.500444 | 0.504733 | down | 0.898683471 |  |
| LAIR1 | 2.159122 | 2.075977 | up | 0.208573382 |  |
| KIR3DL1 | 0.062147 | 0.056613 | up | 0.056271988 |  |
| TMIGD2 | 0.29922 | 0.276175 | up | 0.550990813 |  |
| LGALS9 | 3.177904 | 3.139594 | up | 0.532809514 |  |
| CD28 | 0.87783 | 0.863456 | up | 0.966057681 |  |
| TNFSF15 | 0.776232 | 0.764839 | up | 0.552577459 |  |
| BTLA | 0.48416 | 0.407361 | up | 0.153664563 |  |
| TNFRSF4 | 1.617289 | 1.563254 | up | 0.422178456 |  |
| TNFRSF18 | 2.610694 | 2.576588 | up | 0.684177958 |  |
