# Supplementary material for: Predicting breast cancer prognosis based on a novel pathomics model through CHEK1 expression analysis using machine learning algorithms
Source: PLoS One. 2025 May 9;20(5):e0321717. doi: 10.1371/journal.pone.0321717 (PMC12064205; doi:10.1371/journal.pone.0321717)
Supplement: S4 Table — (DOCX) [file pone.0321717.s016.docx]

**Immune Cell Infiltration Differences Between the PS-high and PS-low Groups**

| Gene | High | Low | Diff. | P | Sig. |
| --- | --- | --- | --- | --- | --- |
| NK | 0.137108 | 0.157834 | down | 1.45E-07 | *** |
| Gamma_delta | 0.112474 | 0.090063 | up | 2.18E-09 | *** |
| CD8_naive | 0.00308 | 0.003703 | down | 1.04E-05 | *** |
| Th17 | 0.005273 | 0.006205 | down | 0.003044 | ** |
| Monocyte | 0.085996 | 0.092705 | down | 0.027045 | * |
| Macrophage | 0.102293 | 0.094614 | up | 0.027396 | * |
| CD8_T | 0.096201 | 0.101681 | down | 0.045455 | * |
| iTreg | 0.005514 | 0.00478 | up | 0.015757 | * |
| Th1 | 0.003631 | 0.003153 | up | 0.024187 | * |
| Central_memory | 0.004124 | 0.00348 | up | 0.012998 | * |
| Effector_memory | 0.000454 | 0.000208 | up | 0.013708 | * |
| DC | 0.096353 | 0.101611 | down | 0.155195 |  |
| Bcell | 0.081257 | 0.080659 | up | 0.99091 |  |
| Neutrophil | 0.098779 | 0.097039 | up | 0.717316 |  |
| CD4_T | 0.083108 | 0.078439 | up | 0.179976 |  |
| NKT | 0.106398 | 0.105339 | up | 0.605376 |  |
| CD4_naive | 0.001361 | 0.001382 | down | 0.522755 |  |
| Tr1 | 0.008241 | 0.007279 | up | 0.050903 |  |
| nTreg | 0.007177 | 0.006594 | up | 0.119637 |  |
| Th2 | 0.006787 | 0.00678 | up | 0.801587 |  |
| Tfh | 0.006916 | 0.006238 | up | 0.106121 |  |
| Cytotoxic | 0.006582 | 0.006463 | up | 0.871908 |  |
| Exhausted | 0.006277 | 0.006768 | down | 0.057917 |  |
| MAIT | 0.007092 | 0.007563 | down | 0.052323 |  |
