# Supplementary material for: Predicting breast cancer prognosis based on a novel pathomics model through CHEK1 expression analysis using machine learning algorithms
Source: PLoS One. 2025 May 9;20(5):e0321717. doi: 10.1371/journal.pone.0321717 (PMC12064205; doi:10.1371/journal.pone.0321717)
Supplement: S5 Table — Tissue Microarray. (DOCX) [file pone.0321717.s017.docx]

**Baseline Characteristics of Patients in the PS-low and PS-high Groups Using the BrC. Tissue Microarray**

| **Variables** | **Total  (n = 89)** | **HS-low  (n =39)** | **HS-high  (n = 50)** | **p** |
| --- | --- | --- | --- | --- |
| **Age, n (%)** | | | | |
| ＜60 | 50 (56) | 24 (62) | 26 (52) | 0.494 |
| ≥60 | 39 (44) | 15 (38) | 24 (48) |  |
| **ER Status, n (%)** | | | | |
| Negative | 26 (29) | 11 (28) | 15 (30) | 0.999 |
| Positive | 63 (71) | 28 (72) | 35 (70) |  |
| **PR Status, n (%)** | | | | |
| Negative | 34 (38) | 18 (46) | 16 (32) | 0.253 |
| Positive | 55 (62) | 21 (54) | 34 (68) |  |
| **HER2 Status, n (%)** | | | | |
| Negative | 62 (70) | 28 (72) | 34 (68) | 0.878 |
| Positive | 27 (30) | 11 (28) | 16 (32) |  |
| **KI67, n (%)** | | | | |
| >14% | 67 (75) | 30 (77) | 37 (74) | 0.945 |
| ≤14% | 22 (25) | 9 (23) | 13 (26) |  |
| **Histologic Grade, n (%)** | | | | |
| G1/G2 | 67 (75) | 30 (77) | 37 (74) | 0.945 |
| G3 | 22 (25) | 9 (23) | 13 (26) |  |
| **Pathologic Stage, n (%)** | | | | |
| I / II | 66 (74) | 31 (79) | 35 (70) | 0.441 |
| III / IV | 23 (26) | 8 (21) | 15 (30) |  |
